# Identification of YES1 Inhibitors From Approved Drugs Using a Multimodal Deep Learning Framework

**DOI:** 10.1002/minf.70061

**Published:** 2026-10-14

**Authors:** Takayoshi Kawabata, Tatsuaki Takeda, Yuta Tanaka, Soichiro Ushio, Akira Miyamoto, Koki Nakagomi, Tomonori Sakai, Risa Maruyama, Takashi Makita, Hirofumi Hamano, Shinichi Toyooka, Yoshito Zamami

**Affiliations:** ^1^ Department of Clinical Pharmacy Graduate School of Medicine, Dentistry and Pharmaceutical Sciences Okayama University Okayama Japan; ^2^ Department of Pharmacy Okayama University Hospital Okayama Japan; ^3^ Center for Education in Medicine and Health Sciences, Graduate School of Medicine Dentistry and Pharmaceutical Sciences Okayama University Okayama Japan; ^4^ Department of Pharmaceutical Sciences for Health Crisis Management Faculty of Pharmaceutical Sciences Fukuoka University Fukuoka Japan; ^5^ Department of Pharmacy Yokohama Municipal Citizen’s Hospital Yokohama Japan; ^6^ Digital Innovation Section Digital Transformation Promotion Department Nippon Shinyaku co., Ltd. Kyoto Japan; ^7^ Department of Pharmacy Medical Development Field Okayama University Okayama Japan; ^8^ Graduate School and Faculty of Information Science and Electrical Engineering Kyushu University Fukuoka Japan; ^9^ Department of General Thoracic Surgery and Breast and Endocrinological Surgery Graduate School of Medicine, Dentistry and Pharmaceutical Sciences Okayama University Okayama Japan

**Keywords:** antitumor agents, drug repositioning, drug resistance, HER2 resistance, virtual screening

## Abstract

While anti‐human epidermal growth factor receptor 2 (HER2) drugs, including trastuzumab improve the prognosis of patients with HER2‐positive cancers, acquired resistance by cancer cells remains a significant clinical challenge. We had previously reported that YES1 is overexpressed in trastuzumab‐resistant breast and lung cancer cells, and that its inhibition restores trastuzumab sensitivity. However, no approved drug specifically targets YES1. In this study, as a drug repositioning approach aimed at rapid clinical application, we constructed a machine learning model capable of learning the interactions between compounds and target proteins and conducted a screening of approved drugs. The strategy identified chlorpromazine and raloxifene as hit compounds. In vitro validation demonstrated that they effectively inhibited YES1 activation at 15 μM after 48 h. The findings suggested that the identified approved drugs might represent promising therapeutic options for overcoming anti‐HER2 drug resistance.

## Graphical abstract

To overcome anti‐HER2 drug resistance, we developed a multimodal deep learning model to identify YES1 inhibitors from approved drugs. By integrating molecular graphs, physicochemical properties, and protein sequence data, our computational screening identified chlorpromazine and raloxifene. Subsequent in vitro validation confirmed their efficacy in inhibiting YES1 activation, suggesting the potential of a drug‐repositioning approach for rapid clinical application.

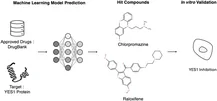

## 1 Introduction

The human epidermal growth factor receptor 2 (HER2) is a receptor tyrosine kinase belonging to the ERBB family. It forms either homodimers or heterodimers and activates the downstream PI3K‐AKT and MAPK pathways to regulate cell division, proliferation, and apoptosis [1]. Overexpression of HER2 is known as an oncogenic driver. It is observed not only in breast cancer but also in a wide range of carcinomas, including gastric, colorectal, lung, and salivary gland cancers [2, 3, 4, 5, 6, 7].

The introduction of anti‐HER2 drugs, including trastuzumab, has significantly improved therapeutic outcomes for HER2‐positive cancers. Particularly in early‐stage HER2‐positive breast cancer, combination therapy consisting of chemotherapy and two anti‐HER2 agents has demonstrated high efficacy, achieving a 3 year invasive survival rate exceeding 90% [8].

However, HER2‐positive cancers have been reported to acquire resistance to anti‐HER2 drugs, leading to disease progression [3, 9]. The acquired resistance poses a significant clinical challenge. Multiple mechanisms of resistance have been reported, including mutations in the HER family, masking or deletion of HER2 epitopes, and activation of alternative pathways [10]. Therefore, strategies to overcome the resistance based on these mechanisms are urgently required.

We had previously reported that YES proto‐oncogene 1 (YES1), a member of the SRC family kinases (SFKs), is overexpressed in the HER2‐positive breast and lung cancer cell lines resistant to trastuzumab, lapatinib, and neratinib [11, 12]. Furthermore, we demonstrated that inhibition of YES1 restores sensitivity to these drugs [11, 12]. YES1 signaling has been reported to contribute to resistance against epidermal growth factor receptor‐tyrosine kinase inhibitors (EGFR‐TKIs) in non‐small‐cell lung cancer [13]. The findings suggested that YES1 is a potential therapeutic target for overcoming resistance to various classes of anticancer drugs across different cancer types.

Among approved drugs, some TKIs, such as dasatinib, exhibit YES1 inhibitory activity. However, their use in combination with anti‐HER2 therapies to overcome drug resistance may be challenging because of their adverse effects, including cardiotoxicity, and their high cost. In recent years, compounds such as CH6953755 and eCF506 have been developed with high selectivity for specific SFKs, including YES1 and SRC [14, 15]. In particular, eCF506 is undergoing phase I clinical trials, and its development is progressing toward regulatory approval. However, the clinical development of these compounds requires considerable time before regulatory approval and carries the risk of previously unrecognized toxicities.

Next, as a drug repositioning approach aimed at rapid clinical application, we constructed a deep‐learning model to screen the approved drugs for YES1 inhibitory activity. Furthermore, we validated the YES1 inhibitory activity of the selected candidate drugs through in vitro assays.

## 2 Materials and Methods

### 2.1 Data Collection and Preprocessing

Bioactivity data (IC_50_ and % inhibition) for SFKs were obtained from the ChEMBL database (version 34) [16]. Only assays meeting the following criteria were retained: standard relation type of “=”, target organism of *Homo sapiens*, and assay type “B” (binding).

Chemical structures were standardized using RDKit (version 2024.09.4). SMILES strings were canonicalized, and for mixtures or salt forms, only the largest molecular fragment was retained. Compounds with invalid SMILES or missing atomic information were excluded from further analysis.

To construct a binary classification model, continuous activity data were converted into inhibitor and noninhibitor classes using thresholds reported in previous binary classification studies [17]. For IC_50_ data, compounds with IC_50_ values below 10 µM were labeled as inhibitors, whereas those with IC_50_ values above 50 µM were labeled as noninhibitors; compounds in the intermediate range were excluded to reduce ambiguity arising from experimental variability. For % inhibition data measured at 10 µM, compounds showing more than 50% inhibition were classified as inhibitors, while those showing less than 25% inhibition were classified as noninhibitors; intermediate values were excluded for the same reason mentioned above.

Duplicate records corresponding to the same compound were further curated. For IC_50_ measurements, duplicate entries were retained only if the difference in pIC_50_ values was less than 0.1, in which case the mean value was used for labeling; otherwise, the data were excluded due to low reliability [17, 18]. For % inhibition data, compounds were retained only when all reported measurements were consistently assigned to the same activity class; conflicting entries were removed.

After preprocessing, a total of 5,165 compounds were retained and used for model training and evaluation (Table S1). The number of compounds along with the distribution of activity classes for the ten targeted SFKs is summarized in Table S2.

### 2.2 Molecular and Target Representations

We constructed a multimodal deep learning model that integrated molecular graph representations, physicochemical descriptors, and target protein representations. Molecular features were extracted using a Graph Convolutional Network (GCN) [19] and Mordred descriptors [20], while protein representations were obtained using the pretrained protein language model ESM‍‐‍2 [21].

#### 2.2.1 Molecular Graph Construction

Canonical SMILES strings were converted into undirected molecular graphs, with atoms and chemical bonds defined as nodes and edges, respectively. The molecular graphs were encoded using a GCN.

Node features included atomic number, aromaticity, formal charge, number of radical electrons, and number of hydrogen atoms as numerical features, along with atom symbol, degree, and hybridization encoded using one‐hot vectors. Bond type was used as the edge feature. The node and edge features were used as inputs to the GCN encoder to generate molecular graph representations.

#### 2.2.2 Molecular Descriptors

To complement the structural representations captured by molecular graphs, physicochemical properties were represented using Mordred descriptors [20]. A total of 1613 two‐dimensional descriptors were calculated for each compound.

Descriptors containing calculation errors or exhibiting a missing rate greater than 30% were removed. Missing values in the remaining descriptors were imputed using the median value of the training dataset. To mitigate multicollinearity, one descriptor from each pair with a Pearson correlation coefficient exceeding 0.9 was removed.

Feature selection was performed using the Boruta algorithm [22], a Random Forest–based wrapper method that identifies statistically significant features by comparing the importance of original features against shadow features. The selected descriptors were subsequently normalized to a range of 0–1 using Min–Max scaling before being input into the model.

#### 2.2.3 Protein Representation

The study focused on ten SFKs, namely, FRK, HCK, LYN, BLK, LCK, FGR, SRMS, FYN, SRC, and YES. Amino acid sequence for each protein were retrieved from the UniProt database [23].

rotein representations capturing evolutionary and structural context were obtained using the pretrained protein language model ESM‐2 (esm2_t33_650M_UR50D) [21]. Each amino acid sequence was input into the model, and mean pooling was applied to the output of the final hidden layer to generate a fixed‐length 1280‐dimensional vector for each protein.

### 2.3 Multimodal Deep Learning Model

#### 2.3.1 Strategy for Handling Data Scarcity

In this study, we aimed to construct a classification model to predict the inhibitory activity against YES1. Nevertheless, the available activity data for YES1 in ChEMBL was limited to 135 entries, which was insufficient for training a deep learning model. Therefore, we adopted a strategy to expand the scope to include SFKs, which share high homology and are structurally and functionally closely related to YES1 [24]. This resulted in a total of 5165 data points for training. By incorporating target protein representations into the model, we aimed to capture the similarities among SFKs and improve prediction robustness for YES1, for which the available data are limited.

#### 2.3.2 Network Architecture

We constructed a multimodal model integrating three different modalities, namely, molecular graph representations, physicochemical descriptors, and target protein representations. Features extracted from each modality were processed via multilayer perceptrons within their respective encoders and compressed into 128‐dimensional vectors.

• Graph encoder: A GCN was employed to extract the local structural features from molecular graphs. Outputs of the GCN layers were aggregated using mean pooling and subsequently transformed by an MLP to generate a 128‐dimensional molecular graph representation.
• Descriptor encoder: While GCNs are effective in capturing local structural patterns, global physicochemical properties are not always explicitly represented. To complement this information, the selected Mordred descriptors were processed using a MLP to obtain a 128‐dimensional physicochemical representation.
• Target encoder: Protein embedding vectors derived from ESM‐2 were processed using an MLP to generate a 128‐dimensional target protein representation.

Rectified Linear Unit activation functions and dropout layers were applied after each linear transformation, except for the final layer of each encoder. The three 128‐dimensional vectors were concatenated and input into a prediction head consisting of three fully connected layers to predict the probability of inhibitory activity.

### 2.4 Model Training and Evaluation

#### 2.4.1 Training Protocol

BCEWithLogitsLoss was used as the loss function and Adam was adopted as the optimization algorithm. To prevent overfitting, early stopping was introduced to terminate training if the validation loss did not improve for 5 consecutive epochs. Random search was used to optimize the hyperparameters, including learning rate, batch size, number of GCN layers, hidden layer dimensions, dropout rate, and weight decay.

#### 2.4.2 Evaluation and Ablation Study

To evaluate the generalization performance of the model, we performed stratified 5‐fold cross‐validation. Considering the imbalanced nature of the dataset, we used the receiver operating characteristic (ROC) and precision‐recall (PR) curves for evaluation, with the area under the curve (AUC) for each being adopted as primary metrics beyond accuracy. Furthermore, to verify the contribution of each modality (structural information, physicochemical descriptors, and target protein information), the following four model variants were constructed and compared:

• GME Model: GCN + Mordred + ESM (all modalities integrated).
• GM Model: GCN + Mordred (without protein information).
• GE Model: GCN + ESM (without descriptors).
• ME Model: Mordred + ESM (without graph structure).

To ensure fair comparison, hyperparameter optimization was performed individually for each variant. For the final screening, the best model demonstrating the highest PR‐AUC in cross‐validation, along with its optimal hyperparameters, was selected.

### 2.5 Drug Screening for YES1 Inhibitors

#### 2.5.1 Screening Library and Preprocessing

To identify drugs that inhibit YES1 from a library of approved drugs, compounds registered in the DrugBank database (version 5.1.10) [25] were selected for screening. As with the training data, preprocessing involved canonicalization of SMILES strings, and for data containing mixtures or salts, only the largest fragment was retained. Compounds included in the training dataset were excluded to ensure fair evaluation.

Following the structural standardization, molecular descriptors were calculated using Mordred. To maintain consistency with the training data, data were cleaned by converting calculation errors, missing outputs, and infinite values into NaNs. The missing values were then imputed using the median values of the corresponding features from the training dataset. Finally, the selected features were scaled using the prefitted scaler derived from the training data. The preprocessing parameters were derived exclusively from the training dataset and applied to the screening library to prevent data leakage and maintain a strict learn‐predict separation [26, 27].

#### 2.5.2 Applicability Domain (AD) Filtering

Tanimoto similarity was used to define the AD of the constructed model. Morgan fingerprints (radius 2, 2048 bits) were calculated for each compound [28], and the maximum similarity with the compounds in the training data was computed. In this study, compounds with a maximum Tanimoto similarity of 0.6 or higher were considered within the model’s AD and subjected to screening.

#### 2.5.3 Prediction and Selection

Compounds passing the AD filtering were predicted using the best model selected in Section 2.4.2. Since the model requires a compound‐target protein pair as input, the target protein input was fixed to the “YES1 ESM embedding vector,” and pairs of each compound and YES1 were fed into the model. Compounds with a calculated prediction probability of 0.5 or higher were selected as candidate compounds exhibiting YES1 inhibitory activity.

### 2.6 In Vitro Validation of Predicted YES1 Inhibitors

#### 2.6.1 Cell Culture and Reagents

The HER2‐positive human breast cancer cell line BT‐474 (#HTB‐20; American Type Culture Collection, Manassas, VA, USA) was used. The trastuzumab‐resistant subline, BT‐474‐R, was established by treating parental BT‐474 cells with increasing concentrations of trastuzumab (ranging from 0.1 to 40 µg/mL) over a period of 10 months [29]. In the established BT‐474‐R cells, overexpression of YES1 was confirmed, and it was reported to be one of the factors contributing to resistance to anti‐HER2 therapy [11]. BT‐474‐R was maintained in Dulbecco’s Modified Eagle’s Medium (high glucose) (#08457‐55; Nacalai Tesque, Kyoto, Japan) supplemented with 10% fetal bovine serum (35‐079‐CV; Corning, New York, NY, USA) and 1% penicillin‐streptomycin solution (×100) (#168‐23191; FUJIFILM Wako Pure Chemical Corporation, Osaka, Japan). The cultures were incubated at 37 °C in a humidified atmosphere containing 5% CO_2_. D‐PBS(‐) (#14249‐24; Nacalai Tesque, Kyoto, Japan) was used for washing the cells during subculturing and sample preparation. For in vitro validation of the model’s predictions, the candidate compounds identified through virtual screening—chlorpromazine hydrochloride and raloxifene hydrochloride (purity >95% by high performance liquid chromatography (HPLC))—were purchased from Tokyo Chemical Industry (Tokyo, Japan). eCF506, used as a control compound, was purchased from Selleck Biotechnology (S0791, Tokyo, Japan).

#### 2.6.2 Cell Viability Assays

The effect of hit compounds on cell proliferation was verified to determine the appropriate treatment concentrations for subsequent experiments. Selected compounds were prepared in 8‐step 2‐fold serial dilutions, starting from 100 µM, and added to the cells. After 72 h of incubation, cell viability was measured using the Cell Counting Kit‐8 (#343‐07623; Dojindo, Kumamoto, Japan) to calculate the IC_50_ values. Furthermore, to determine whether the antitumor activity of the hit compounds was attributable to YES1 overexpression in the resistant cells, cell viability was also measured in parental BT‐474 cells using the same procedure, and the IC_50_ values were calculated. eCF506 was used as a reference compound for selective YES1 inhibition, and cell viability was evaluated using the same assay procedure. The concentration range of eCF506 was adjusted based on its inhibitory activity.

#### 2.6.3 Western Blot Analysis

Cells treated with the predicted drugs were lysed in RIPA buffer (P0278; Sigma–Aldrich, St. Louis, MO, USA) supplemented with Phosphatase Inhibitor Cocktails 2 and 3 (P5726 and P0044; Sigma–Aldrich, St. Louis, MO, USA) and cOmplete, Mini Protease Inhibitor Cocktail (11836153001; Sigma–Aldrich, St. Louis, MO, USA). The lysates were centrifuged at 20 000× g for 15 min at 4 °C, and the supernatant was collected therefrom. Protein concentrations were determined using the DC Protein Assay Kit (Bio‐Rad Laboratories, Hercules, CA, USA). All protein recovery procedures were performed on ice.

Equal amounts of protein samples were separated by SDS‐PAGE using 12% Mini‐PROTEAN TGX Precast Gels (#4561045/#4561046; Bio‐Rad Laboratories, Hercules, CA, USA) in running buffer diluted from 10x Tris/Glycine/SDS Buffer (#1610732; Bio‐Rad Laboratories, Hercules, CA, USA). Proteins were then transferred to 0.2 µm PVDF membranes (Trans‐Blot Turbo Transfer Pack; #1704156; Bio‐Rad Laboratories, Hercules, CA, USA) using the Trans‐Blot Turbo Transfer System. The membranes were blocked with SuperBlock T20 Blocking Buffer (#37536; Thermo Fisher Scientific, Carlsbad, CA, USA) for 30 min at room temperature.

Primary antibodies were diluted in Can Get Signal Immunoreaction Enhancer Solution (Solution 1; #NKB‐101; Toyobo, Osaka, Japan) and incubated with the membranes for 1 h at room temperature. The primary antibodies used included Phospho‐Src Family (Tyr416) Rabbit Monoclonal Antibody (#59548S, 1:1000; Cell Signaling Technology, Danvers, MA, USA), Yes Rabbit Monoclonal Antibody (#65890S, 1:1000; Cell Signaling Technology, Danvers, MA, USA), and β‐Actin Rabbit Monoclonal Antibody (#8457S, 1:5000; Cell Signaling Technology, Danvers, MA, USA). Actin served as a loading control.

Subsequently, the membranes were incubated for 1 h at room temperature with Anti‐rabbit IgG, HRP‐linked Antibody (#7074, 1:10000; Cell Signaling Technology, Danvers, MA, USA) diluted in Can Get Signal Immunoreaction Enhancer Solution (Solution 2; #NKB‐101; Toyobo, Osaka, Japan). Protein bands were visualized using ECL Prime Western Blotting Detection Reagent (#RPN2232; Cytiva, Marlborough, MA, USA) and imaged with the ChemiDoc Touch Imaging System (Bio‐Rad Laboratories, Hercules, CA, USA).

### 2.7 Statistical Analysis

All statistical analyses were performed using R, version 4.6.1. Differences in cell viability between BT‐474 and BT‐474‐R cells at each drug concentration as well as differences in IC_50_ values between the two cell lines were assessed using Welch’s t‐test. A *p*‐value <0.05 was considered statistically significant.

## 3 Results

### 3.1 Model Performance and Contribution of Modalities

To evaluate the contribution of each modality to the prediction of YES1 inhibitory activity, four model variants (GME, GM, GE, and ME) were constructed based on the combinations of molecular graph representation (G: GCN), molecular descriptors (M: Mordred descriptors), and protein representation (E: ESM embedding). The performance of these models was compared using stratified 5‐fold cross‐validation. The results are shown in Table 1, and the ROC and PR curves for each model are shown in Figure 1. Among the evaluated models, the GE model achieved an ROC‐AUC of 0.911 ± 0.006 and a PR‐AUC of 0.869 ± 0.013. The GME model showed similar performance, with an ROC‐AUC of 0.910 ± 0.007 and a PR‍‐‍AUC of 0.866 ± 0.009. The ME model exhibited slightly lower performance, with an ROC‐AUC of 0.894 ± 0.016 and a PR‐AUC of 0.845 ± 0.022. In contrast, the GM model exhibited lower performance than the other models, with an ROC‍‐‍AUC of 0.838 ± 0.022 and a PR‐AUC of 0.783 ± 0.032. The results suggested that incorporating protein representation could contribute to improved prediction performance for YES1 inhibitory activity. Furthermore, the comparable performance of both GME and GE models indicated that combining structural and protein information alone can sufficiently achieve predictive accuracy equivalent to that using all modalities and that the additional inclusion of molecular descriptors would provide only limited improvement in this dataset. Based on these results, the GE model was selected for subsequent screening. To further characterize the protein representations used in the model, we compared the ESM‐2 embeddings of the SFKs. The ESM‐2 embeddings showed high similarity among the SFKs, including YES1 (Table S3).

**TABLE 1:** Ablation study evaluating the contribution of each modality to model performance.

Model features	ROC_AUC	PRC_AUC	Accuracy
GE^a^	0.911 ± 0.006	0.869 ± 0.013	0.835 ± 0.011
GME^b^	0.910 ± 0.007	0.866 ± 0.009	0.843 ± 0.007
ME^c^	0.894 ± 0.016	0.845 ± 0.022	0.822 ± 0.016
GM^d^	0.838 ± 0.022	0.783 ± 0.032	0.748 ± 0.017

a GCN + ESM.

b GCN + Mordred + ESM.

c Mordred + ESM.

d GCN + Mordred.

**FIGURE 1:**
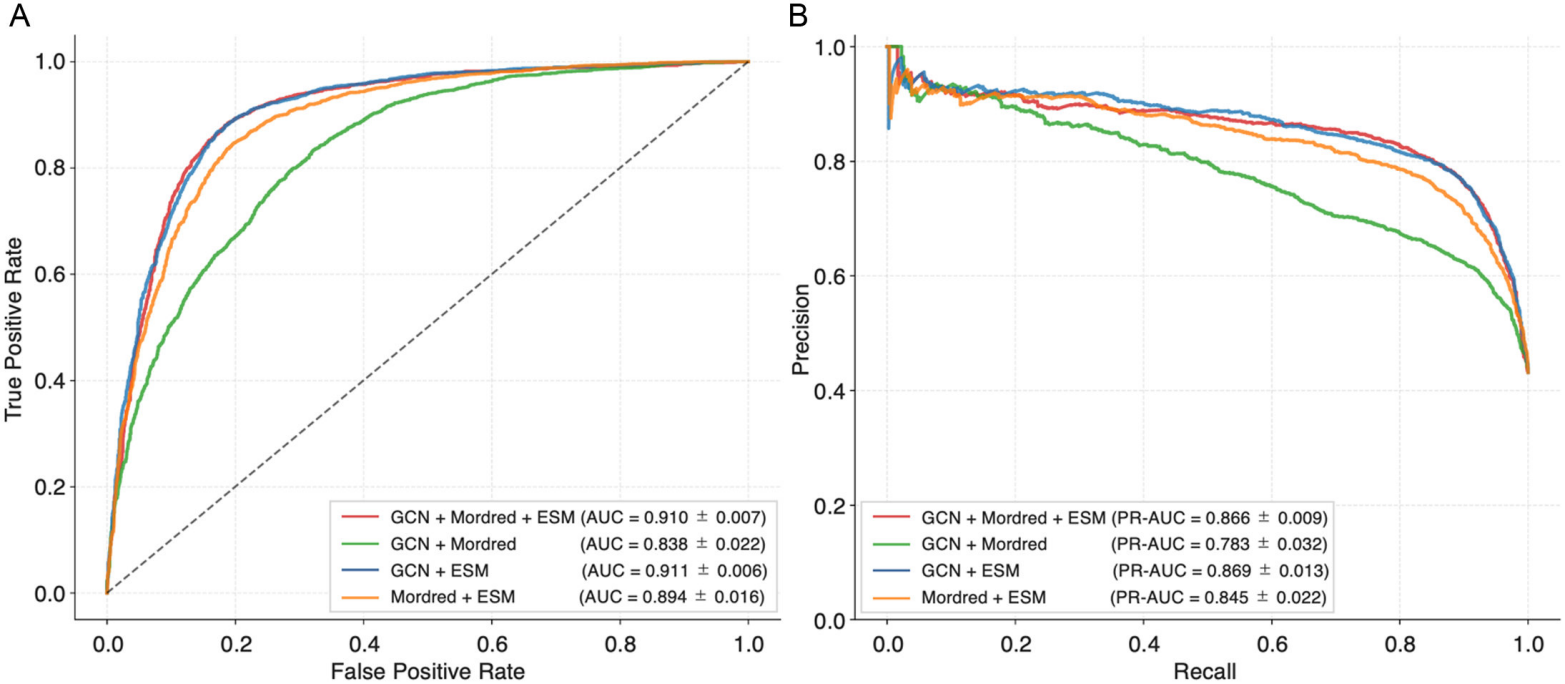
Receiver operating characteristic and precision‐recall curves of the four model variants.

### 3.2 Identification of Candidate YES1 Inhibitors via Drug Repositioning

To identify the approved drugs with potential YES1 inhibitory activity, a screening analysis was conducted using drugs registered in DrugBank (version 5.1.10; total entries: 2587). The compound selection workflow used in the screening is shown in Figure 2A. First, preprocessing steps were applied, including SMILES normalization and retention of the largest fragment. Next, compounds with known activity values against YES1 that were already included in the training dataset were removed. Conversely, compounds that had activity data only for other SFKs were retained since these compounds could provide useful structural information related to kinase inhibition for model learning. As a result of these procedures, 10 compounds were excluded. Subsequently, filtering based on the AD was performed. A comparison of the chemical space between the training dataset (ChEMBL‐derived compounds) and the DrugBank compounds used for screening revealed differences in their distributions (Figure 2B). Therefore, for each DrugBank compound, the Tanimoto similarity with all compounds in the training dataset was calculated, and the distribution of the maximum similarities is shown in Figure 2C. After AD filtering, 102 compounds remained. The compounds were then screened using the GE model. A total of 10 compounds were identified as candidates with potential YES1 inhibitory activity (Table 2). Among these, five compounds were known tyrosine kinase inhibitors (TKIs). Of the remaining five compounds, three were either not approved by the FDA or had been withdrawn from the market. Therefore, chlorpromazine and raloxifene were selected for subsequent experimental validation of YES1 inhibitory activity.

**FIGURE 2:**
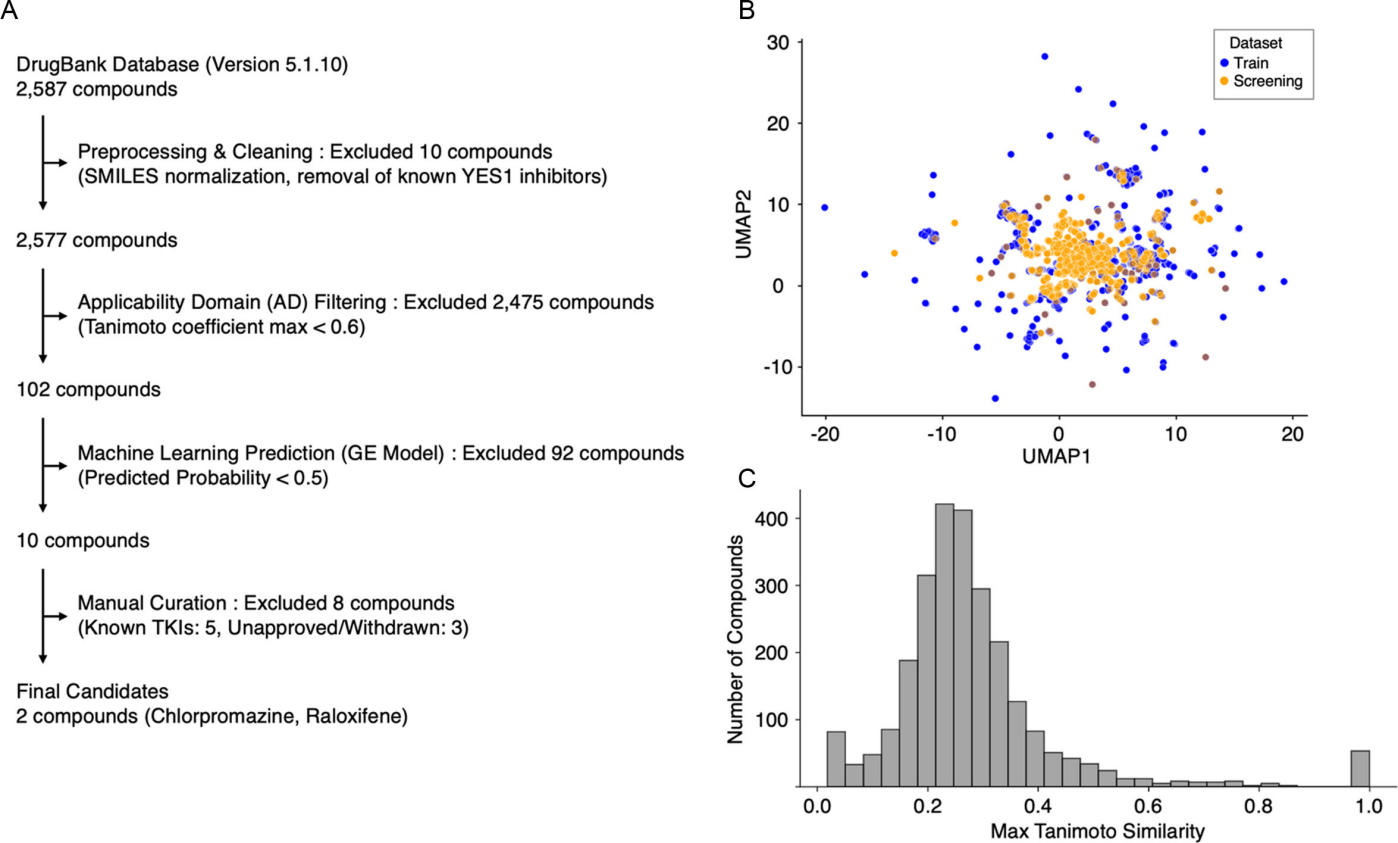
Workflow and chemical space analysis of DrugBank screening for YES1 inhibitors. (A) Stepwise screening workflow of DrugBank compounds. After preprocessing and removal of the known YES1 inhibitors, applicability domain (AD) filtering based on Tanimoto similarity (threshold ≥ 0.6) was applied, followed by prediction using the GE model. Compounds with predicted probability ≥ 0.5 were retained, and manual curation was performed to exclude the known TKIs and unapproved/withdrawn drugs. (B) UMAP visualization of the chemical space of training data (blue) and DrugBank screening compounds (orange). (C) Distribution of maximum Tanimoto similarity between each DrugBank compound and the training dataset.

**TABLE 2:** Top‐ranked candidate compounds from DrugBank predicted to inhibit YES1 by the GE model.

Generic name	Probability	FDA
Chlorpromazine	0.968	Approved
Imatinib	0.955	Approved
Gefitinib	0.911	Approved
Triflupromazine	0.899	Not approved
Dacomitinib	0.866	Approved
Afatinib	0.835	Approved
Nilotinib	0.630	Approved
Promazine	0.588	Discontinued
Raloxifene	0.586	Approved
Arzoxifene	0.541	Not approved

### 3.3 Experimental Validation of YES1 Inhibitory Activity in Cancer Cells

To validate whether the selected compounds, chlorpromazine and raloxifene, exhibit YES1 inhibitory activity in cells, we evaluated their effects in BT‐474 and BT‐474‐R cells.

First, cytotoxicity against BT‐474‐R cells was evaluated, and IC_50_ values were calculated. In BT‐474‐R cells, chlorpromazine and raloxifene showed IC_50_ values of 9.22 ± 0.36 μM and 16.46 ± 0.78 μM, respectively. The corresponding IC_50_ values in parental BT‐474 cells were 14.42 ± 0.36 μM and 28.74 ± 1.48 μM, respectively. The IC_50_ values were significantly lower in BT‐474‐R cells than in parental BT‐474 cells for both chlorpromazine and raloxifene (Table S4).

Next, cell viability was compared between parental BT‐474 and BT‐474‐R cells. The results are presented in Figure 3A,B. Chlorpromazine and raloxifene significantly reduced cell viability in BT‐474‐R cells compared with parental BT‐474 cells at concentrations of 12.5 and 25 μM. We then analyzed western blotting results to assess the inhibitory effects of the compounds on YES1 phosphorylation. To evaluate the concentration‐ and time‐dependent effects, we assessed concentration gradient (5, 10, and 15 μM) and temporal alteration (6, 24, 48, and 72 h) effects. Specifically, we evaluated the effects of concentrations of 5, 10, and 15 μM after 72 h of treatment (Figure 3C,D). Moreover, changes over time were observed following treatment with 15 μM of each compound (Figure 3E,F). Our results revealed that neither compound affected total YES1 protein expression levels remarkably. However, YES1 phosphorylation levels decreased time‐ and concentration‐dependently. Specifically, chlorpromazine exhibited YES1 inhibition at concentrations of at least 10 μM, while raloxifene exerted inhibitory effects starting from 5 μM. Furthermore, both drugs were inhibitory from 48 h onward. These findings indicated that both compounds reduced YES1 phosphorylation without altering total YES1 protein levels in BT‐474‐R cells. For comparison, the results of cell viability assays in BT‐474 and BT‐474‐R cells using eCF506 as a reference compound are presented in Figure S1, while the results of the western blot analysis evaluating YES1 inhibition are presented in Figure S2. eCF506 exhibited YES1 inhibitory activity at lower concentrations than chlorpromazine and raloxifene.

**FIGURE 3:**
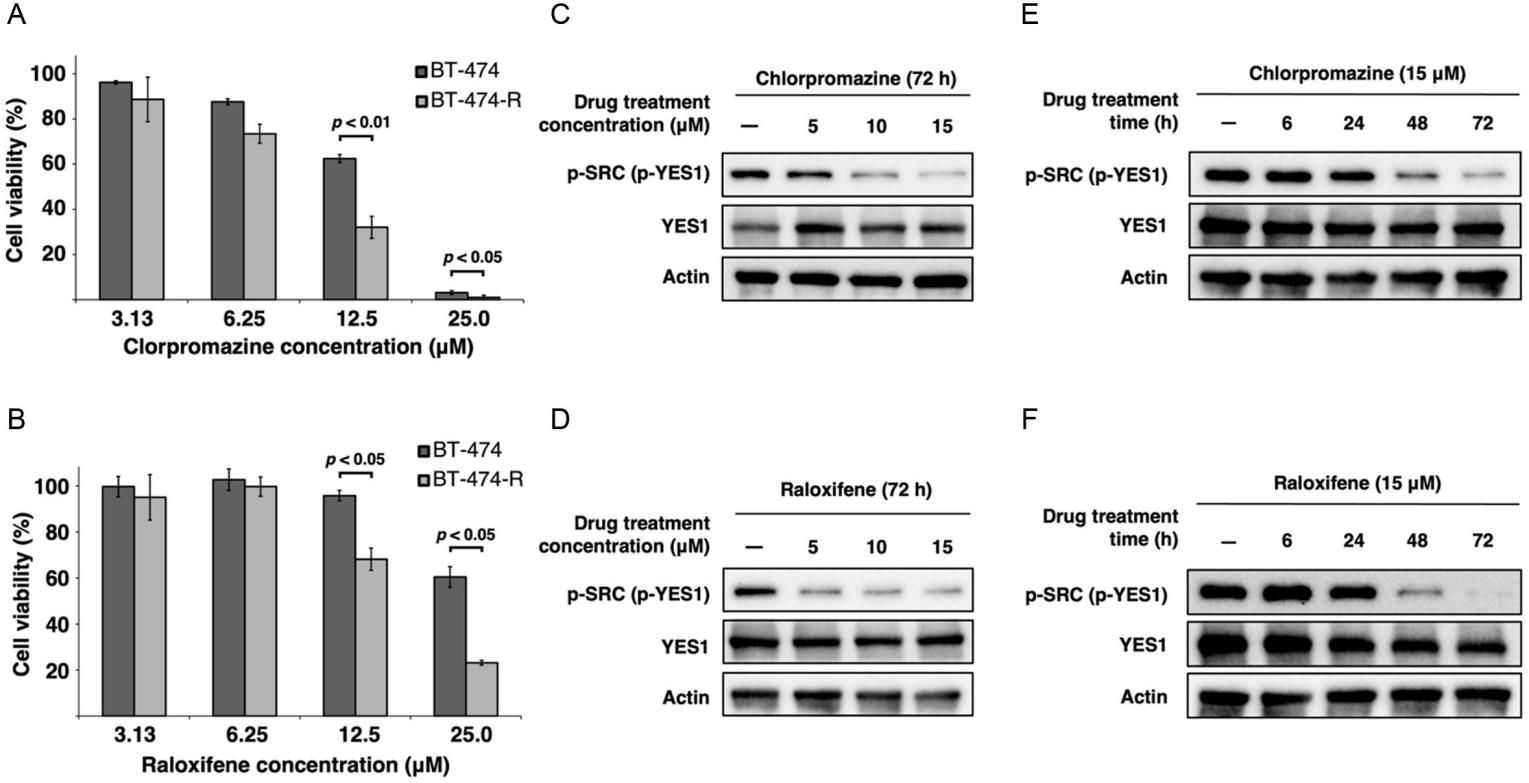
Cell viability and western blot analysis of YES1 phosphorylation following treatment with chlorpromazine and raloxifene. (A,B) Cell viability of BT‐474 and BT‐474‐R cells at the indicated concentrations of (A) chlorpromazine and (B) raloxifene after 72 h of treatment. Experiments were performed independently 3 times, and data are presented as the mean ± standard error. Statistical significance was assessed using Welch’s *t*‐test. (C,D) Concentration‐dependent changes of (C) chlorpromazine and (D) raloxifene in p‐SRC (p‐YES1) levels at 72 h. (E,F) Time‐dependent changes in p‐SRC (p‐YES1) levels of (E) chlorpromazine (15 μM) and (F) raloxifene (15 μM). DMSO concentration was maintained at 0.1% for all conditions, including that of the vehicle control.

## 4 Discussion

We constructed a classification model to predict the YES1 inhibitory activity and screened the compounds registered in DrugBank. The hit compounds identified through this screening demonstrated inhibitory effects on YES1 phosphorylation, suggesting their potential as candidates for drug repositioning.

One of the challenges in constructing a prediction model for YES1 inhibitors is the scarcity of bioactivity data. Indeed, only 135 activity records for YES1 were available in the ChEMBL database (Table S2). For such data‐poor targets, integrating activity data from closely related targets and simultaneously incorporating both target protein and compound features into the model can improve predictive performance, as shown in previous studies [30]. Furthermore, even among the structurally and functionally related kinases, such as SFKs, the integration of data from related targets has been suggested to contribute to improved predictive performance [31]. However, when integrating data across multiple targets, a compound may exhibit activity against only a specific target. Without explicitly incorporating target protein information into the input, this could lead to contradictory training data within the model. Therefore, the integration of target protein information is a key step in model construction.

SFKs share a high degree of homology among their members, making it necessary to accurately distinguish subtle differences between the kinases. To incorporate sequence information of the target proteins as features, we introduced the protein language model ESM‐2 [21]. In our ablation study, the model lacking target protein information (GM) showed reduced predictive performance (ROC‐AUC: 0.838). The reduction is likely attributable to the model’s limited ability to distinguish the differences in compound selectivity across targets. Models integrating target information via ESM‐2 (GME and GE) exhibited improved performance, with the ROC‐AUC increasing from 0.838 to over 0.910. Although SFKs share high sequence similarity (Table S3), integrating ESM representations could enable the model to capture the subtle sequence‐level differences across the kinases. The results suggested that incorporating protein information into the model could facilitate the discrimination of activity differences within the SFK family.

Furthermore, the effect of adding manually designed Mordred descriptors to the GCN as compound features was limited (Table 1). A recent large‐scale benchmark study reported that learning based on graph convolutions achieves representational power and predictive accuracy comparable to or better than models using classical physicochemical descriptors or fingerprints [32]. Our results were consistent with these findings, suggesting that the molecular graph representation by the GCN could sufficiently capture the structural information of the compounds. Additionally, an improvement in predictive performance was observed by integrating protein representations from ESM alongside the molecular graph representations. GCN can capture chemical structural features, such as local structural patterns and functional group arrangements, directly from molecular graphs [33]. Meanwhile, the protein embeddings obtained from ESM function as feature representations that reflect the evolutionary and structural information inherent in amino acid sequences [21]. By integrating the two types of information, the proposed model may have learned latent patterns associated with ligand‐target binding, similar to previous studies, rather than merely relying on compound structural similarities [34].

To investigate whether the antitumor effects of chlorpromazine and raloxifene identified through screening were associated with YES1 inhibition, cell viability was compared between parental BT‐474 and BT‐474‐R cells. Cell viability was significantly reduced in the resistant cells compared with the parental cells at specific concentration ranges (Figure 3A,B). Furthermore, the western blot results showed that the concentrations at which YES1 inhibition was observed (10 μM for chlorpromazine and 5 μM for raloxifene) were close to the concentration ranges that reduced cell viability in the resistant cells (Figure 3C,D). These findings suggest that chlorpromazine and raloxifene may have selectively reduced cell viability in the resistant cells, potentially by inhibiting YES1 phosphorylation.

In recent years, eCF506 has been developed as a highly selective inhibitor for specific SFKs, including YES1 and SRC. eCF506 is undergoing Phase I clinical trials. According to previously reported data, the IC_50_ of eCF506 in cell‐based assays ranges from 15 to 220 nM [35]. eCF506 has been structurally modified to maximize selectivity for its target kinases [15] and exhibits lower IC_50_ values than those of chlorpromazine (IC_50_: 9.22 μM) and raloxifene (IC_50_: 16.46 μM). Furthermore, based on the results of the YES1 inhibition assays (Figures 3C–F and S2), eCF506 exhibited substantially greater inhibitory potency against YES1 than chlorpromazine and raloxifene. One possible explanation for the relatively modest inhibitory activity of chlorpromazine and raloxifene is that the screening library consisted of approved drugs that were not TKIs. These drugs were originally optimized for other biological targets and thus lacked structural features specifically tailored for kinase inhibition [33]. In this study, to ensure prediction reliability by restricting the screening compounds to the chemical space represented in the training dataset, an AD defined by a Tanimoto similarity threshold of 0.6 with the training compounds was applied, thereby restricting the screening candidates. The non‐TKI‐approved drugs identified from compounds passing the AD filtering exhibited inhibitory effects on YES1 phosphorylation, consistent with the model prediction. The results supported that the model predictions have a certain level of reliability within the defined AD. Overall, the relatively weak inhibitory activity might reflect the identification of previously unrecognized pharmacological effects from an existing drug library that was not designed for kinase inhibition.

Another possible explanation is the heterogeneity of the ChEMBL‐derived activity data. Public bioactivity datasets are known to contain measurements obtained from different research groups and experimental conditions, which inevitably introduce variability [36]. Chiba et al. had reported that when integrating multiple data sources without incorporating assay conditions into the model, prediction accuracy may decrease [31]. In the present study, diverse data sources were integrated, and a classification threshold was applied, and such experimental noise may have contributed to reduced accuracy in activity prediction. Despite the experimental challenges, by applying an AD to reduce false positives, the study successfully identified chlorpromazine and raloxifene as hit compounds with YES1 inhibitory activity. Although the weaker YES1 inhibitory activity of chlorpromazine and raloxifene compared with selective YES1 inhibitors represents a limitation, these drugs are widely used in clinical practice as an antipsychotic and a selective estrogen receptor modulator, respectively, and their safety profiles are well‐established. Furthermore, several studies have reported the potential anticancer activities of these drugs [37, 38, 39]. Although eCF506 has advanced to phase I clinical trials, the clinical development period from phase I to regulatory approval has been reported to take approximately 7 years on average [40], and the development of novel compounds carries the risk of discontinuation due to previously unrecognized toxicities. Therefore, chlorpromazine and raloxifene may offer an advantage for rapid clinical translation because they are already approved for clinical use. The identification of their YES1 inhibitory activity suggested them to be particularly effective in treating cancers characterized by high YES1 expression.

This study has several limitations regarding target selectivity, which may be related to the high similarity observed among SFKs, including YES1, in the ESM‐2 embedding space. Even eCF506, which exhibits high selectivity for YES1 and SRC, has been reported to inhibit multiple SFKs [15]. Although we implemented strategies in the machine learning model to select compounds with YES1 inhibitory activity, the public datasets used for model training do not contain comprehensive activity data against all SFKs. Therefore, we cannot exclude the possibility that chlorpromazine and raloxifene also inhibit other SFKs. Future studies should focus on further investigating these compounds, including detailed selectivity evaluations across the SFK panel and structural development to improve activity, in order to verify their YES1 inhibitory effects at more physiological concentrations and their contribution to restoring anti‐HER2 sensitivity.

In conclusion, the multimodal model combining deep learning‐based feature extraction methods—namely, GCN and protein language model ESM‐2—demonstrated favorable predictive performance. By establishing an AD to suppress false positives, the constructed model identified novel compounds that inhibit YES1 phosphorylation from existing drugs with established safety profiles. The approved drugs identified in this study have the potential to serve as novel therapeutic options for overcoming resistance to both anti‐HER2 drugs and EGFR‐TKIs.

## Author Contributions

**Takayoshi Kawabata:** conceptualization, methodology, software, formal analysis, investigation, resources, writing – original draft preparation, visualization. **Tatsuaki Takeda:** conceptualization, methodology, validation, formal analysis, investigation, resources, data curation, writing ‍–‍ original draft preparation, project administration, funding acquisition. **Yuta Tanaka:** writing – review, and editing. **Soichiro Ushio:** methodology, data curation, writing – review, and editing. **Akira Miyamoto:** methodology, data curation, writing – review, and editing. **Koki Nakagomi:** methodology, data curation, writing – review, and editing. **Tomonori Sakai:** validation, writing – review, and editing. **Risa Maruyama:** methodology, data curation, writing – review, and editing. **Takashi Makita:** writing – review and editing, supervision. **Hirofumi Hamano:** software, writing – review and editing. **Shinichi Toyooka:** writing – review and editing, supervision. **Yoshito Zamami:** writing – review and editing, supervision, funding acquisition. All authors gave final approval to the submitted manuscript. **Tatsuaki Takeda** is responsible for the overall content as guarantors.

## Funding

This work was supported by the Japan Society for the Promotion of Science (23K14412, 25K02867, and 25K22708).

## Conflicts of Interest

The authors declare no conflicts of interest.

## Supporting information

Supplementary Material

Supplementary Material

## Data Availability

The data that support the findings of this study are available from the corresponding author upon reasonable request.
