# Supplementary material for: Identification of YES1 Inhibitors From Approved Drugs Using a Multimodal Deep Learning Framework
Source: Mol Inform. 2026 Oct 14;45(10):e70061. doi: 10.1002/minf.70061 (PMC13665583; doi:10.1002/minf.70061)
Supplement: Supplementary file 1 [file MINF-45-e70061-s001.pdf]

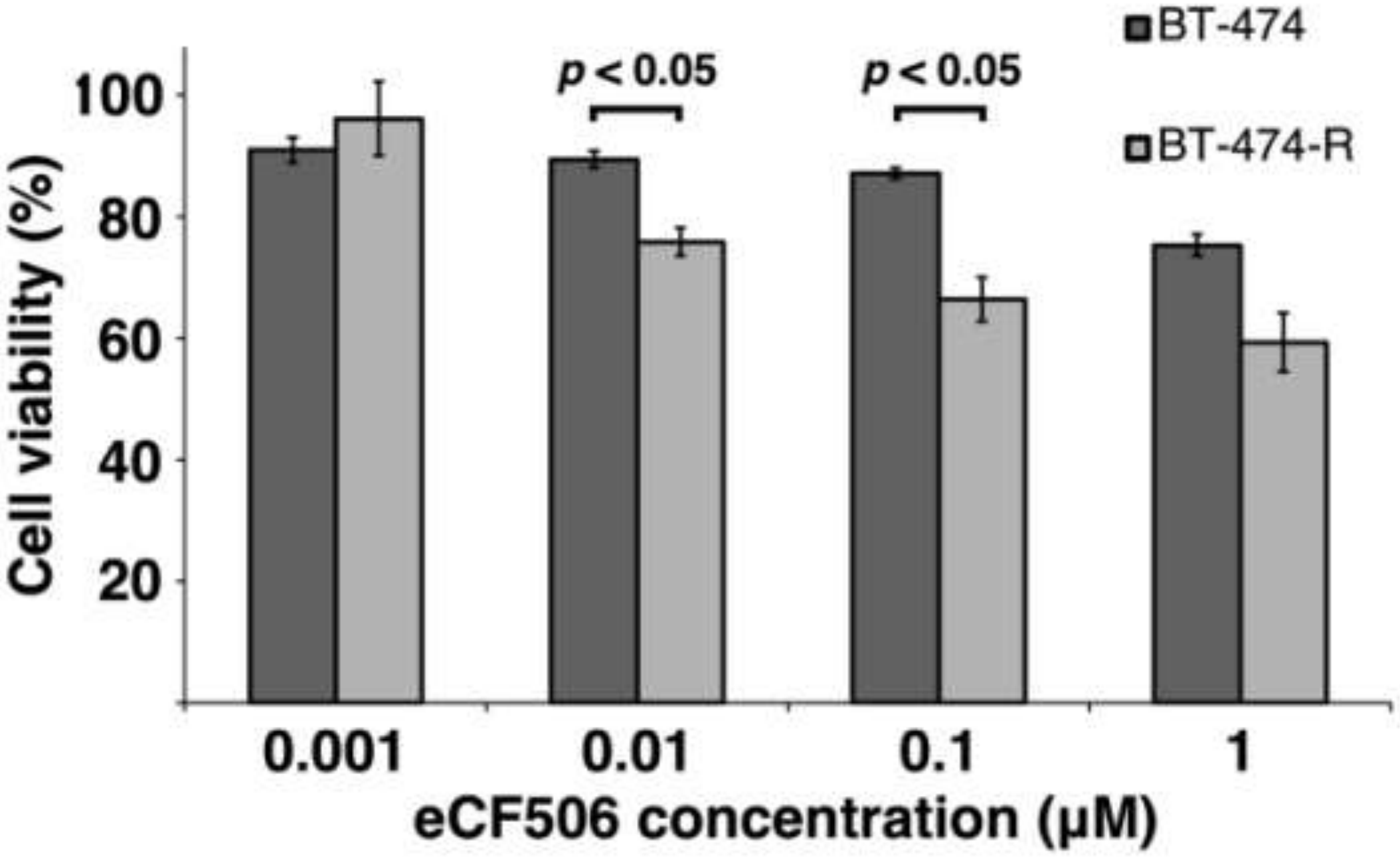


**Figure S1**. Cell viability of BT-474 and BT-474-R cells following treatment with eCF506 at the indicated concentrations for 72 h. Experiments were performed independently three times, and data are presented as the mean ± S.E.M. Statistical significance was determined using Welch's t-test for comparisons of cell viability between BT-474 and BT-474-R cells at each drug concentration.

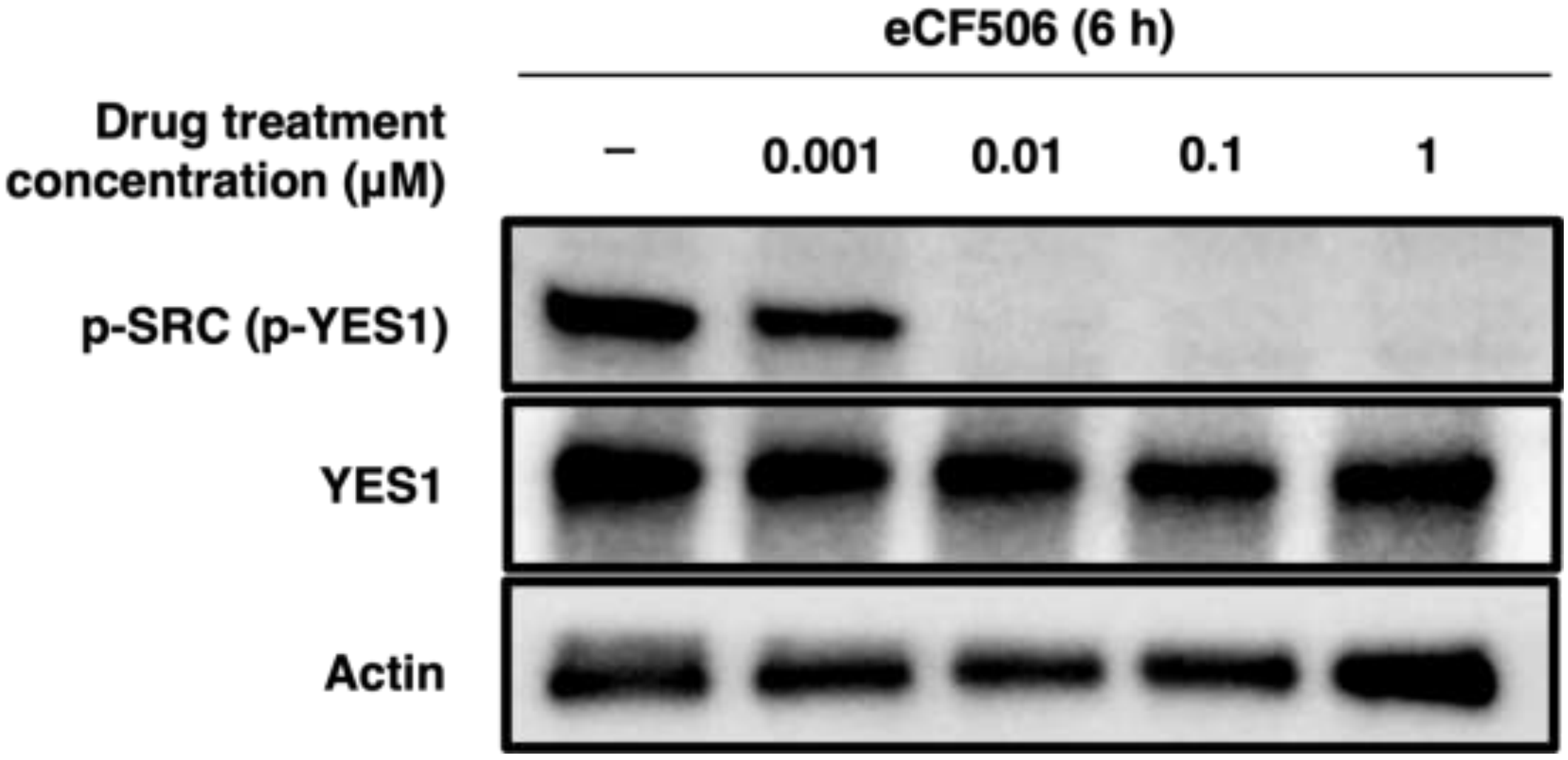


**Figure S2**. Western blot analysis of YES1 phosphorylation following treatment with eCF506 in BT-474-R cells. Concentration-dependent changes at 6 h. DMSO concentration was maintained at 0.1% for all conditions, including that of the vehicle control.
